# An Ultrasonic Multiple-Access Ranging Core Based on Frequency Shift Keying Towards Indoor Localization

**DOI:** 10.3390/s150818641

**Published:** 2015-07-30

**Authors:** Laurent Segers, David Van Bavegem, Sam De Winne, An Braeken, Abdellah Touhafi, Kris Steenhaut

**Affiliations:** 1 Department of Industrial Sciences and Technology (INDI), Vrije Universiteit Brussel, Pleinlaan 2, Elsene 1050, Belgium; E-Mails: david.van.bavegem@vub.ac.be (D.V.B.); sam.de.winne@vub.ac.be (S.D.W); an.braeken@vub.ac.be (A.B.); abdellah.touhafi@vub.ac.be (A.T.); 2 Department of Electronics and Informatics (ETRO), Vrije Universiteit Brussel, Pleinlaan 2, Elsene 1050, Belgium; E-Mails: ksteenha@etro.vub.ac.be (K.S.)

**Keywords:** indoor ultrasound localization, indoor ultrasound ranging, FPGA ranging, FPGA correlator, ultrasound orthogonal frequency shift keying, ultrasound MEMS

## Abstract

This paper describes a new approach and implementation methodology for indoor ranging based on the time difference of arrival using code division multiple access with ultrasound signals. A novel implementation based on a field programmable gate array using finite impulse response filters and an optimized correlation demodulator implementation for ultrasound orthogonal signals is developed. Orthogonal codes are modulated onto ultrasound signals using frequency shift keying with carrier frequencies of 24.5 kHz and 26 kHz. This implementation enhances the possibilities for real-time, embedded and low-power tracking of several simultaneous transmitters. Due to the high degree of parallelism offered by field programmable gate arrays, up to four transmitters can be tracked simultaneously. The implementation requires at most 30% of the available logic gates of a Spartan-6 XC6SLX45 device and is evaluated on accuracy and precision through several ranging topologies. In the first topology, the distance between one transmitter and one receiver is evaluated. Afterwards, ranging analyses are applied between two simultaneous transmitters and one receiver. Ultimately, the position of the receiver against four transmitters using trilateration is also demonstrated. Results show enhanced distance measurements with distances ranging from a few centimeters up to 17 m, while keeping a centimeter-level accuracy.

## 1. Introduction

Accurate indoor localization opens new perspectives towards location-aware applications. A non-exhaustive list of applications that might take advantage of accurate indoor localization solutions include automation, guidance and assistance of people, virtual reality, *etc*. Examples of automation include indoor cleaning robots; where the robots clean a well-defined area, indoor navigation for automated assistance robots in hospitals and retirement homes. Indoor localization can also be used in combination with other technologies, like smart glasses towards virtual reality applications. Furthermore, the indoor remote guidance of drones could benefit from this technology, since the exact location of the drone against walls can be defined. Other usages include patient tracking in hospitals and the automated guidance of blind people into busy and complex environments. More generally, people can actively benefit from this technology by using it as a guidance system in route retrieval in complex buildings and by using it as an extension of the ubiquitous available Global Positioning System (GPS).

GPS allows one to plan a route to a given destination. Although GPS is a well-established system, the system lacks accurate positioning in buildings. Several systems and implementations have been proposed to alleviate this shortcoming. These systems can be classified by the approach used, the accuracies they can deliver, the real timeness, the required amount of computational capabilities and the cost to implement such systems. The most common techniques used in the literature are based on the time difference of arrival (TDoA). This technique uses two different transmission media, where the fast transmission medium (generally radio communication) is used as a synchronization between devices and a slower transmission medium (e.g., ultrasound) is used for distance ranging.

In the class of TDoA techniques, several topologies have been developed. Some techniques use a rather simple ranging scheme, where an ultrasonic pulse at a given frequency is sent [1,2,3,4]. A hardware-based envelope detector detects the presence of an incoming signal. When a pulse is detected, the receiver is triggered, and an estimation of the ranging is performed. This technique does not involve expensive computational power to achieve indoor localization. However, the ultrasound pulse is prone to in-band noise, which may affect the accuracy of the system. Another approach proposed in the literature makes use of ultrasound signals, which are modulated using orthogonal sequences [5,6,7,8,9]. This technique is commonly referred to as code division multiple access (CDMA). Two major CDMA techniques have been proposed so far. The first one uses the direct sequence spread spectrum approach (DSSS). The DSSS method uses a single carrier on which an orthogonal sequence is modulated. The second approach is referred to as frequency hopping spread spectrum (FHSS) and involves several rapidly-switching frequency carriers on which data are modulated [9]. Both implementations offer a better reliability against noise, but have the major disadvantage of requiring more computational power. Therefore, distance ranging is mostly performed offline, which reduces the possibilities to integrate this technique into real-time and low-power implementations.

In order to alleviate these shortcomings, we implement a novel FHSS ranging approach. Our implementation consists of combining the strengths of the orthogonal ultrasound modulated signal with the computational processing capabilities of field programmable gate arrays (FPGAs). FPGAs offer the possibility to apply parallel processing in a real-time fashion. However, FPGAs are limited in the amount of available resources. Therefore, we optimize the processing of the signal demodulation and ranging in order to fit our system into medium-sized FPGAs. Our new implementation is evaluated using statistical analysis. Besides the computational implementations, a low-power and low-cost hardware solution will be investigated in order to augment the possibilities of integration with other existing systems.

The results show that our system can perform ultrasound ranging within distances from a few centimeters up to 17 m. The overall accuracy remains constant at a level of 1 to 2 cm. The implementation allows one to calculate the distance against four simultaneous transmitters, while keeping the required logic gates of a Spartan-6 XC6SLX45 below 30%. A test case showing the feasibility towards indoor localization with position retrieval onto a map is also demonstrated.

The paper is structured as follows. Section 2 presents some background and related work. In Section 3, we give an brief overview of the implemented system. In Section 4, we focus on the FPGA receiver logic, the mathematical background for adequate signal correlation and ranging and the optimizations performed. In Section 5, the FPGA resource utilization in terms of maximum attainable clock speed and logic requirements are shown. We apply analysis with our new implementation measurements (Section 6), where we determine that accurate ranging up to the centimeter level is possible. Finally, in Section 7, we present our conclusions and present possibilities towards a useful implementation.

## 2. Background and Related Work

Several indoor localization techniques have been implemented and reported in the literature. Some of these techniques are based on the principle of range estimation between two devices. These techniques comprise time of arrival (ToA) [10], time difference of arrival (TDoA) and received signal strength indicator (RSSI) [11,12]. Among other techniques, one can find the angle of arrival (AoA) to estimate the orientation of devices against other devices, which leads to a position estimation [11,13,14]. Within the range estimation techniques, the TDoA technique has been reported to be able to achieve centimeter to sub-centimeter accuracy.

TDoA generally uses two different propagation media in order to estimate the distance between two devices. These two propagation media, mostly radio frequency (RF) combined with an ultrasonic signal (US), have different propagation speeds. This difference in propagation speed is used to calculate the distance between a transmitter and a receiver. Although much research has been done in the field of indoor localization techniques, the TDoA ranging technique remains the most accurate and one of the most promising techniques for indoor localization [1,2,3,4,5,6,7,8,9,15].

Several time difference of arrival methods have been proposed. The Active Bat [1], Dolphin [2], Cricket [3] and iLoc [4] use an ultrasonic pulse in combination with an RF packet to achieve accurate ranging up to centimeter-level ranging. However, since the ultrasonic signal is a pulse, the systems are allowed to have only one active ultrasonic transmitter at a given timeslot. This principle is also known as time division multiple access (TDMA). With an increasing number of transmitters, the refresh rate diminishes, which has led to different timing schemes. Moreover, the use of an ultrasonic pulse at a given frequency reduces the robustness against in-band noise.

Others reported another approach to alleviate these shortcomings by applying code division multiple access (CDMA) [5,6,7,8,9]. CDMA has been proven to be an efficient technique within the field of telecommunications and consists of spreading the data by using orthogonal codes. Depending on the application, FHSS and DSSS are the main techniques developed. The former method spreads the data onto several rapidly-switching carriers, where the switching pattern follows a given orthogonal code. The second method spreads the data with an orthogonal code onto a single carrier. The DSSS method has been implemented by several research teams [5,6,7,8]. The reported accuracies range from a few millimeters to a few centimeters. FHSS has been reported to achieve comparable results [9].

In a previous work, a comparison between DSSS and FHSS has been done [9]. Results have shown that both methods achieve comparable results while only one ultrasonic transmitter is active. When two devices are sending simultaneously, results show that DSSS yields a better accuracy compared to FHSS. However, the experiments also demonstrate that FHSS achieves a better reliability over longer distances compared to DSSS. This can be due to the fact that FHSS uses several carriers on which data are modulated. This modulation scheme makes it less prone to in-band noise, which is the case with DSSS when one transmitter interferes with another transmitter. In [16], FHSS has been simulated against DSSS, whereas in [17], FHSS has been used in combination with AoA. The latter system uses an array of MEMS microphones and calculates the reception angle using the MUSIC algorithm.

The Active Bat, Cricket Dolphin and iLoc use an ultrasonic pulse for distance calculations. The travel time of that pulse gives an estimation of the distance between transmitter and receiver. This rather simple approach can be implemented using conventional micro-controllers. The FHSS and DSSS approaches however, require advanced computational capabilities in order to perform the distance calculations. Several implementations require off-line calculation infrastructure in order to process the ultrasonic data. With the upcoming embedded FPGA-based computational accelerators, these techniques can be applied within the embedded systems. Andy Ward *et al.* have successfully implemented an adaptive despreader technique for the DSSS approach on FPGA using the finger-printing approach [5].

In [9], the authors used an electrostatic transducer for the transmitters. This electrostatic transducer offers a 20 kHz to 100 kHz frequency range and is primarily used to access the broader frequency ranges required. However, the major disadvantages of this transducer are the small beam angle of 15 degrees and the high operation voltages required. These make the electrostatic transducer unsuitable for embedded systems. Other methods mainly use piezocrystal transducers [1,2,3,4,7].

New technologies, like smartphones, have been demonstrated to be capable of sending ultrasonic signals up to a frequency of 22 kHz [18]. The integration of the ubiquitous smartphones alongside with the emergence of MEMS-based sensors within indoor localization opens a new perspective for a variety of applications. Therefore, we will focus our research on ultrasonic indoor localization with the possible inclusion of smartphone technology. We propose a TDoA implementation that makes use of ultrasound CDMA-based signals, with frequencies between 24.5 kHz and 26 kHz. In order to demodulate such signals at lower calculation costs and in a real-time fashion, we implement a spread spectrum demodulator onto an FPGA. We also choose MEMS technology for receiving together with piezoelectric crystals for sending ultrasound signals. Both transducers are low cost, offer the requested frequency range and have proven their capabilities in similar implementations. The next sections provide a detailed overview of our implementation, ranging from the transducer level up to the FPGA implementation.

## 3. System Description

**Figure 1:**
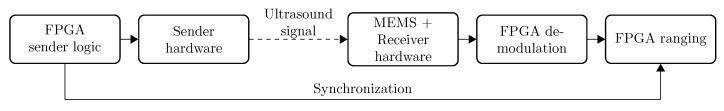
Setup of our experiments. For simplicity, only one transmitter and one receiver are represented in the Figure 1.

Our ultrasonic ranging system is composed of one receiver (Figure 1) and multiple transmitters. The transmitters emit an orthogonal ultrasonic coded signal, which is received and processed by the receiver. Both transmitters and receiver are equipped with an embedded FPGA, which has two major purposes. On the one hand, it facilitates the generation of the orthogonal ultrasonic signal, while on the other hand, due to the high degree of parallel processing capabilities, the FPGA enables the real-time embedded processing of the orthogonal ultrasonic signals. Transmitters and receiver are synchronized by means of a wire, which minimizes the synchronization error during the experiments. Both the transmitters and the receiver are equipped with additional hardware, which consists of a piezoelectric crystal for the transmitters, a MEMS transducer for the receiver and the interfacing hardware between the transducers and the FPGAs. The different parts of our orthogonal ultrasonic ranging method will be detailed in the following paragraphs. One should note that for simplicity, only one transmitter will be used in the system description of the following sections. Adding transmitters to this system is done by duplicating the transmitter system and its synchronization wire.

### 3.1. Hardware

Both the transmitter and receiver are equipped with transducers capable of sending and receiving ultrasonic signals. At the receiver side, we choose to implement two different topologies based on MEMS transducers.

In the first receiver topology, we use the omnidirectional analog SPU0410LR5H-QB MEMS transducer from Knowless [19]. The transducer offers a usable frequency range between 20 kHz and 30 kHz. The output signal is amplified with a factor of 1000 and filtered by hardware in order to allow only the desired frequency range. The signal is then compared to a given threshold and converted into a binary signal. This method enables one to convert analog sinusoidal waves into square waves, where the upper side of the wave represents a logical “1” and the lower side a logical “0”. After this conditioning, the signal is processed by the FPGA. The FPGA platform chosen is the Atlys board provided by Digilent. This board consists of a Xilinx Spartan 6 XC6SLX45 FPGA running at a clock speed of 100 MHz [20]. Although this method eliminates the need for an analog-to-digital converter (ADC), the receiver loses the ability to clearly identify multiple simultaneous transmitters.

For the second receiver topology, the comparator has been changed with an ADC. The ADC that we use is the 10-bit ADC101S021 from Texas Instruments [21] with a sampling rate set at 100 kS/s. The ADC can be interfaced through a serial communication, which is compatible with several standards, such as the serial peripheral interface (SPI). The 10-bit samples are then processed by the FPGA. With a 10-bit sample width resolution, the principle of superposition can remain. This allows the system to identify several simultaneous transmitters.

At the transmitter side, we choose the Prowave 250ST180 piezocrystal as the transmitters’ transducer [22]. This ultrasonic transducer is capable of producing sound waves in a frequency range of 24 kHz to 26 kHz. The transducer offers a large beam angle of 90 degrees with operation voltages up to 20 Vrms. These properties make this transducer much more suited for embedded applications than the electrostatic counterparts. Moreover, this operating frequency range matches the frequency range proposed by the smartphone implementation [18] and the chosen SPU0410LR5H-QB MEMS transducer.

### 3.2. Frequency Shift Keying Modulation

**Figure 2:**
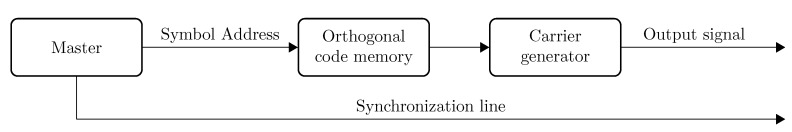
FPGA transmitter logic.

In [9], FHSS has been used in combination with orthogonal codes in order to estimate the range between two devices. When several transmitters are simultaneously transmitting, the receiver is able to distinguish the streams from the different transmitters by filtering the incoming ultrasonic signal. In the FHSS modulation scheme, data are modulated alongside an orthogonal sequence onto several given frequency channels. In our implementation, the data are omitted (*i.e*., always equal to “1”), which turns the FHSS modulation scheme into a frequency shift keying (FSK) modulation scheme where the data are given by the orthogonal code itself. The modulator of the transmitter is implemented onto an FPGA, and is composed of three components. The first component is the frequency generator. Depending on the input data, this component generates a predefined frequency. The code for the FSK scheme is stored in the code memory. This code memory issues a new code symbol when a given address is applied. This address is bounded by the length of the orthogonal code. The last component is the master module. The master module controls the transmitter logic and issues an incrementing symbol address at regular time intervals. When all symbols are issued, the master module pauses the system during a given amount of time and pulls a synchronization line to zero. After this pause, the master restarts the process, and the synchronization line is raised to “1”. This process is repeated every time as long as the system is enabled. Figure 2 gives an overview of the implemented transmitter logic. All of the modules developed can be adapted to match any needs. The amount of carrier frequencies, the baud rate (*i.e*., time between two consecutive frequency hops) and the length of the orthogonal code are parametrized in order to allow more flexibility during the design and implementation phase.

### 3.3. FSK Demodulation

**Figure 3:**
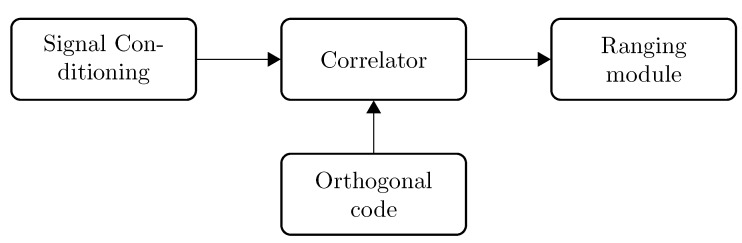
Range estimation.

An FHSS demodulation scheme has been proposed in [9]. The proposed demodulation scheme is partially based on work that has been achieved within ultrasonic ranging with DSSS. The system is composed of a picoscope, which acts as an analog-to-digital converter, a set of bandpass finite impulse response (FIR) filters, a correlator and a synchronization module. All operations are performed off-line on a computer. The samples are stored in a file and processed with a MATLAB script. This technique requires computational capabilities that are typically not available on embedded systems. Our novel approach consists of porting the complete calculation chain onto an FPGA-based calculator. This FPGA calculator correlates the incoming ultrasonic signal against the desired orthogonal code and delivers a distance estimation together with a correlation quality, which is proportional to the length of the correlator. The FSK demodulation and ranging implementation is composed of three major modules: the signal conditioning, the signal correlator and the ranging module. These modules are detailed in the following subsections. A general overview of the system is given in Figure 3.

## 4. Ranging Implementation

### 4.1. Signal Conditioning

The signal conditioning is implemented using two different methods. The first implementation uses a simplified methodology, which makes use of the first hardware topology. The digital signal provided by the hardware removes the necessity of an ADC interface, and the ultrasonic signal is directly processed by the FPGA. The incoming signal is processed by a frequency finder, which allows detecting the availability of a signal in a given frequency channel. The output of the frequency finder is composed of a set of one-bit signals, where each signal reflects the availability of a signal in each frequency channel. Although this method has the advantage of simplifying the approach for the analog-to-digital conversion, this implementation uses a one-bit signal from the hardware. Information about signal superposition is lost, and thus, this method allows one to track only one transmitter at a given time.

**Figure 4:**
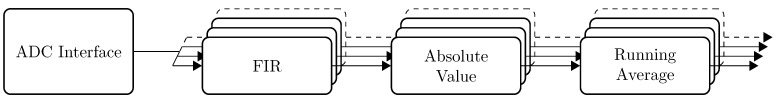
Signal conditioning based on the FIR filter approach. The output of the running average is processed by the signal correlator.

In the second topology, the analog signal is converted to a digital signal using an ADC. This ADC provides a 10 bit-wide signal, which is processed by the FPGA. The signal is first filtered by a series of parallel FIR bandpass filters. Each filter is centered around a frequency channel and has an order of 64. This filter order offers enough steepness to efficiently discriminate the available frequency channels. The absolute value is taken from the output of the FIR filters, and a running average smooths the signals. This results in a certain signal level when a signal is detected or zero when no signal has been processed. Figure 4 depicts this approach.

The output of the running average is composed of *n* bit-wide buses, where each bus represents one frequency channel. When a signal is present, the samples reach a value closer to the maximum, while an empty signal is represented by values close to zero.

### 4.2. Signal Correlation

After the signal conditioning, the correlator module tracks the incoming signal for the highest probability of orthogonal code occurrence. Equation (1) represents the discrete time domain correlation of two signals *f* and *g*.

(1) $$\left(f*g\right)\left[n\right]=\sum_{m=-\infty }^{\infty }f\left[n+k\right]\cdot g\left[k\right]$$

The correlation operation for the ranging method can be summarized as follows:

(a) Prepare the orthogonal sequence.
(b) Shift the samples one position against the orthogonal sequence.
(c) Multiply all of the samples with their corresponding orthogonal code value.
(d) Sum all of the obtained values.
(e) Keep track of the time.
(f) When a correlation peak is found, store it with the corresponding time index.
(g) Repeat the operations from (b) to (f) for all of the input samples.

FPGAs offer the possibility to enable parallel processing. This is especially the case for the correlator module, where the multiplications can be done in parallel. The above-described method presumes an infinite amount of samples. However, an FPGA contains a limited amount of logic cells. Therefore, the length of the correlation is limited to a value proportional to the length of the orthogonal code. The orthogonal code is stored in the reference signal buffer, and the samples are correlated against the reference signal. By doing so, the amount of samples stored corresponds to the length of the correlator. When a new sample is available, all of the samples are shifted with one position in the correlator, and a new correlation is recalculated. The correlation also starts from time zero (*i.e*., the initialize timestamp of the FPGA).

The correlation as stated in Equation (1) requires a given amount of multiplications and additions to compute each correlation, where each code symbol is represented as a binary signal (*i.e*., logic “1” or a logic “0”). The multiplication process of each sample (*i*) can therefore be translated into a multiplexer operation (Equation (2)), which results in a value $c_{i}$.

(2) $$c_{i}=\left\{\begin{array}{c} sample(i),codebit=1\\ -sample(i),codebit=0 \end{array}\right.$$

The naive method utilizes two buffers to store the reference signal (*i.e*., the orthogonal code) and the samples. The orthogonal code is expanded so that the complete reference buffer is populated. To facilitate this expansion, the correlator is subdivided into *n* correlation modules. Each module corresponds to the correlation of one orthogonal code symbol, which is in turn subdivided into *m* correlation cells. The total correlation *C* is calculated by summing all of the individual correlations cell results $c_{i}$ (Equation (3)).

(3) $$C=\sum_{i=0}^{i<n\cdot m}\left(c_{i}\right)$$

**Figure 5:**
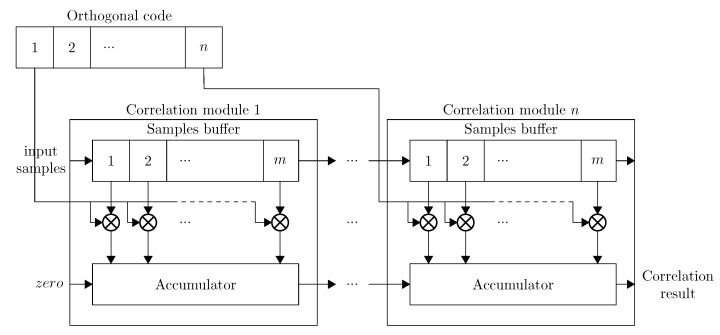
Naive correlator implementation.

The amount of correlation cells *m* is given by the relationship between the bitrate *B* of the signal and the sampling rate $f_{s}$ (Equation (4)). Figure 5 gives an overview of the implementation of the naive correlator.

(4) $$m=\frac{f_{s}}{B}$$

**Figure 6:**
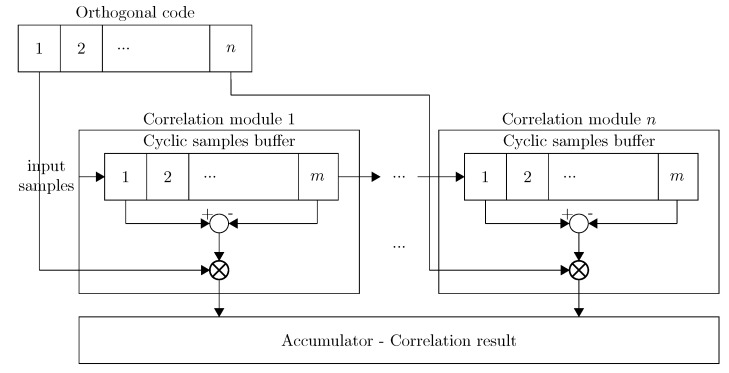
Optimized correlator implementation.

The amount of calculation logic scales linearly with the sampling rate and the amount of code symbols used. Therefore, we propose an optimized design in which we reduce this amount of operations. In the naive method, all correlation modules compute each sample against a given correlation symbol. Each sample is correlated *m* times within each correlator module. Thus, each sample results in the same correlation value as long as it remains in the same correlation module. Redundant calculations can be avoided by storing the samples in a cyclic buffer and by calculating the correlations only on the first and last sample within each module. The intermediate results however need to be stored in an accumulator, so the correlator module can keep track of the total correlation. This is done by adding the correlation to a result register. The correlation of the last element is subtracted from this register when it leaves the correlation module. The total correlation difference ($C_{diff}$) over all *n* correlation modules is given in Equation (5). The amount of calculations is thus reduced to *n* correlation operations. The use of a cyclic buffer enables the VHDL (Very high speed integrated circuit Hardware Description Language) synthesizer to allocate block RAM modules instead of logic gates. A block RAM (BRAM) module is an entity within the FPGA dedicated to fast data storage and retrieval. This approach is demonstrated in Figure 6.

(5) $$C_{diff}=\sum_{i=0}^{i<n}\left\{\begin{array}{c} sample{\left(i\right)}_{in}-sample{\left(i\right)}_{out},codebit\left(m\right)=1\\ -(sample{\left(i\right)}_{in}-sample{\left(i\right)}_{out}),codebit\left(m\right)=0 \end{array}\right.$$

**Figure 7:**
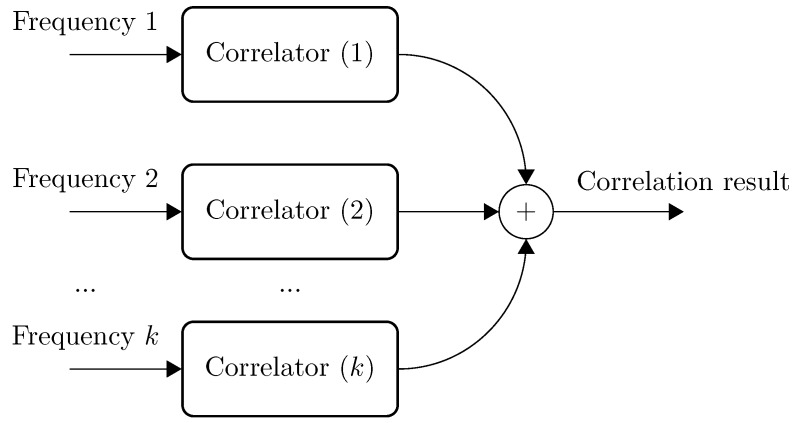
Correlation of multiple (k) frequency channels.

The conditioning modules propose samples of variable precision. The optimized correlator therefore offers a parameterizable bus width for both the input samples and the accumulator result. Aside from the bus width, the conditioning module offers a given amount of FSK frequency channels. Therefore, each frequency channel is theoretically processed by a dedicated correlation chain (Figure 7), where each of the different correlation chains performs the same operation. The results from the different correlation chains are added together. The result is then processed by the timer module.

### 4.3. Correlation Optimizations towards Multiple Simultaneous Transmitters

The proposed optimization from the previous section allows one to reduce the amount of correlation operations involved in the range estimation process between one transmitter and the receiver. Further optimizations can be achieved by calculating the commutative correlations for each frequency channel in a sequential manner (Figure 7) and, thus, to restrict the amount of required logic to only one correlator chain. The counterpart for this operation is an augmentation of the amount of clock ticks involved for computing the multichannel correlations. Equation (6) gives the relationship between the amount of clock ticks ($C_{ticks}$), the length of the orthogonal code (*n*) and the amount of frequency channels ($n_{f}$) used.

(6) $$C_{ticks}=n_{f}\cdot n$$

In our setup, $n_{f}=2$ and n=63, leading to $C_{ticks}=126$. To estimate the distance against several transmitters, one could duplicate the correlator in order to obtain several parallel correlators. However, the amount of required logic to implement such a design would augment linearly with the amount of transmitters. Another approach consists of using additional clock cycles. The correlator computes a new correlation value when a new sample is available. Depending on the implemented system, the time delay between two consecutive samples is given by the ADC interface or the frequency finder.

**Figure 8:**
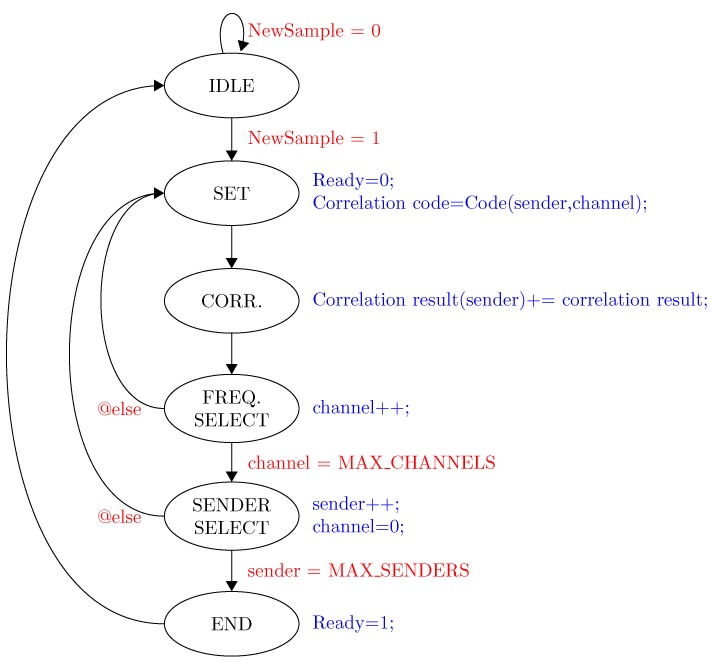
State machine of the correlation controller. Blue labels indicate the state operations, while the red labels refer to conditions between state transitions.

**Figure 9:**
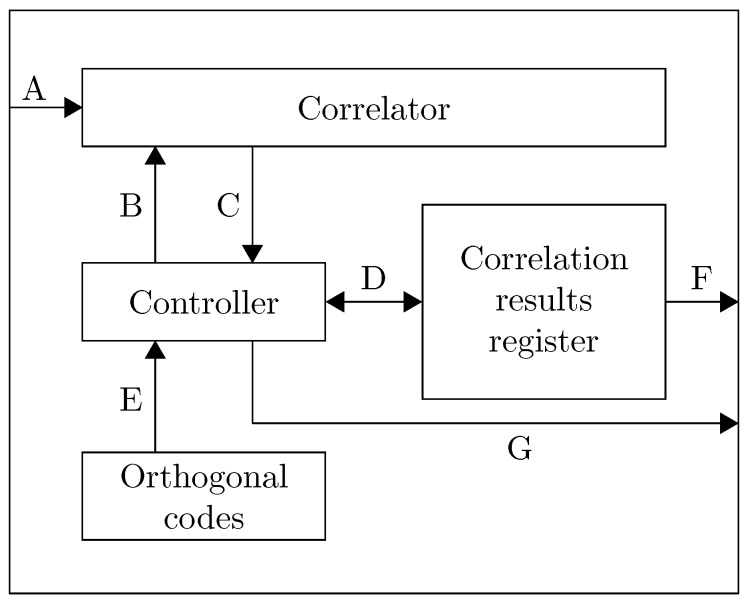
Complete correlation implementation with the controller, the correlator and result registers. The submodules are connected through the input samples (**A**); the code symbols of the current transmitter (**B**); the current correlation result (**C**); the access to the correlation results register of all transmitters (**D**); access to all orthogonal codes (**E**); the correlation results towards the timing module (**F**); and a signal indicating that the correlation is ready (**G**).

In order to implement both optimizations, a state machine is added to the correlator, which controls the data flow of the correlator and registers involved in the correlation against different transmitters (Figure 8). A dedicated control signal allows one to trigger a “ready” signal when all correlations have been processed (Figure 9). The amount of transmitters (*S*) that can be processed within two consecutive samples is given by the sampling speed ($f_{s}$), the FPGA clock speed ($F_{FPGA}$) and the amount of clock ticks required to compute the correlation of one transmitter ($C_{ticks}$) (Equation (7)).

(7) $$S<\frac{F_{FPGA}}{C_{ticks}\cdot f_{s}}$$

The theoretical amount of transmitters that can be processed in our system, can be calculated with the following values:

- $C_{ticks}=126$,
- $F_{FPGA}=100\;MHz$ (Atlys board oscillator frequency),
- $f_{s}=100\;kHz$ (ADC sample frequency).

This leads to a value of S=7.94 transmitters. This value is floored to seven, since the amount of clock ticks involved to calculate the correlations against all transmitters may not exceed the amount of clock ticks between two consecutive samples (*i.e.*, 1000 clock ticks per sample).

### 4.4. Range Estimation

**Figure 10:**
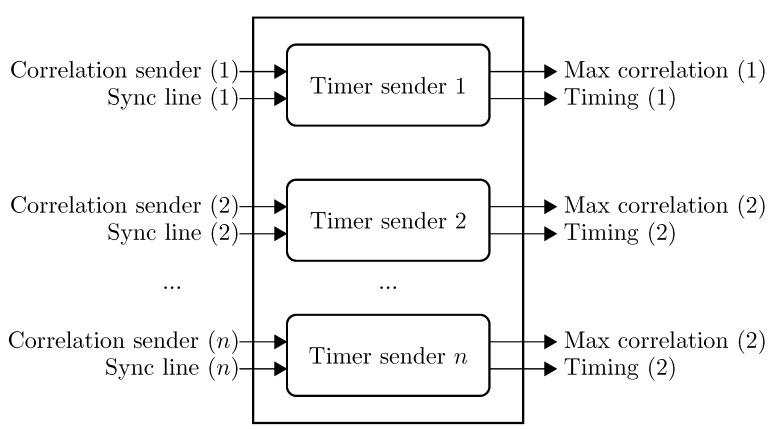
Timing module. Each of the submodules calculates the distance relative to one transmitter and the best achieved correlation.

Distance estimation requires time synchronization, a correlator peak detector and a timer module. Time synchronization enables the devices to have a common time reference between transmitter and receiver, which is used to estimate the time of travel of an ultrasonic signal. In our implementation, we choose to synchronize the devices by means of wires. The transmitter pulls the wire to a logic “1” while sending and to “0” when idle. The receiver synchronizes itself against the transition from “0” to “1”, and the correlator calculates the signal equality against a given orthogonal code. When the signal shows a strong correlation with the code, a correlation peak occurs. The time difference between the time synchronization and the correlation peak is proportional to the distance between transmitter and receiver. In order to keep track of this time difference, a module keeps track of the highest correlation peak time. This module is reset when the receiver is synchronized with the transmitter. In order to track several simultaneous transmitters, this module is duplicated for each transmitter, where each of the transmitters can reset the corresponding module by a dedicated synchronization line. Figure 10 shows the timing module implementation at the receiver’s node for multiple simultaneous transmitters.

## 5. FPGA Resource Utilization

In our experiments, we use the Xilinx Spartan 6 XC6SLX45 FPGA Atlys board provided by Digilent [20]. The FPGA offers 54,576 slice registers, 27,288 slice lookup tables (LUT), 6822 slices and 232 BRAM modules of eight bits. We compare the occupation ratio of the naive correlation method, the optimized method, which allows one to track one transmitter, and the optimized method, which allows one to track four simultaneous transmitters. The first two methods utilize a two bit-wide sample buffer, whereas the last method is implemented using an eight bit-wide sample buffer. All methods are implemented comprising their respective signal conditioning and timing modules. The ranging system is implemented using one to four orthogonal codes (*i.e*., four simultaneous transmitters) with a length of 63 symbols, while two parallel frequency channels are processed by the correlator. Although the theoretical limit of our system is bounded to detect seven simultaneous transmitters, the correlation for four simultaneous transmitters is implemented, since this amount corresponds to the required amount of transmitters for effective localization. In the first method, we choose a sample frequency of 25 kS/s, while in the optimized implementation, a sample frequency of 100 kS/s is chosen. For optimal correlation accuracy, a buffer length for each correlation module of respectively 100 samples and 400 samples is preferred (Equation (4)). All implementations are written in VHDL, are synthesized using the Xilinx ISE tools [23] and have been downloaded onto the Atlys board.

**Figure 11:**
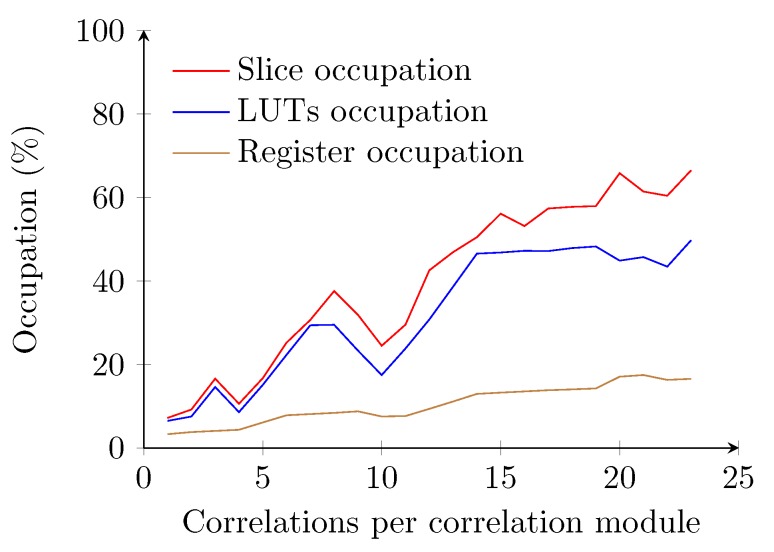
Occupation of the receiver using the naive correlator implementation. The graph covers a range from one correlation per correlation module up to 23 correlations per correlation module.

In the implementation using the naive method, the amount of required logic increases linearly with the amount of samples to be buffered, which agrees with the assumptions from Section 4.2. However, the VHDL synthesizing tools could not implement a system where the correlation modules utilize a sample buffer of a length beyond 23 samples (Figure 11). One of the reasons is that the required amount of interconnections between the FPGA modules overpass the availability of the FPGA. The maximum attainable clock speed for the implementation with a sample buffer length of 20 drops below 50 MHz. Since the amount of required samples to be stored in each correlation module does not reach the minimal requirements, this method will not be implemented for ranging analysis.

**Figure 12:**
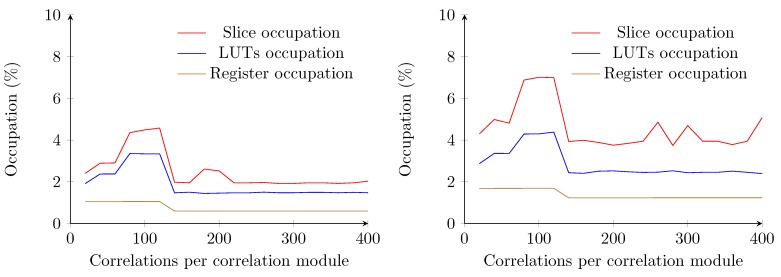
Occupation of the receiver using the optimized correlation implementation with a two bit-wide sample buffer. One transmitter correlation (**left**) and four transmitters correlation (**right**) are shown.

By contrast, using the optimized method with a two bit-wide sample buffer allows one to reduce the occupation ratio to less than 3% of the total FPGA. The amount of required logic gates increases up to a sample buffer length of 120. From a length of 130 samples, the occupation ratio drops and stagnates at values inferior to 3% on average (Figure 12). For lengths inferior to 130, the synthesizer allocates more slices to store the samples, whereas for lengths starting at 130, the BRAM modules of the FPGA are utilized. A total of 63 BRAM modules are required to store the samples for correlations. The length required for each correlation module (*i.e*., 400) is also fulfilled.

By extending this optimized implementation to four transmitters, one can notice that the required amount of logic slightly increases to an average of approximately 5%. This is mainly due to the amount of registers necessary to store the orthogonal codes and the results of the correlations and timing. A decrease in logic occupation is also noticeable from a sample buffer length of 130 (Figure 12). The amount of block RAM required equals the amount required for estimating the distance to one transmitter. The maximum attainable clock speed of the optimized implementations for one transmitter remains constant at 177 MHz, well above the oscillator speed of the Atlys board.

**Figure 13:**
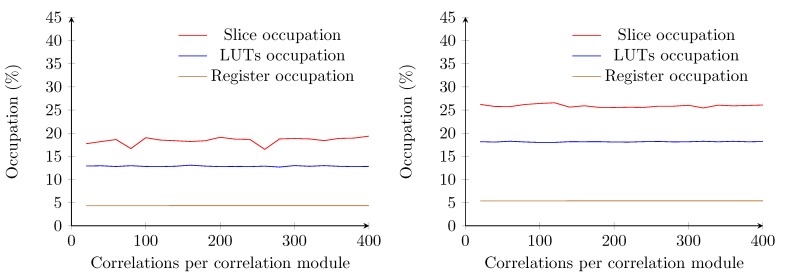
Occupation of the receiver using the optimized correlation implementation with an eight bit-wide sample buffer. One transmitter correlation (**left**) and four transmitters correlation (**right**) are shown.

When the eight bit-wide sample buffer is used, a sample frequency of 100 kS/s is chosen. Since we work with two frequency channels, the total length of the combined samples is 16 bits wide. For this reason, the sample buffer is always implemented in BRAM. The maximum attainable clock speed for this design is 142 MHz, which is lower than the previous implementation, but still above the oscillator speed of the Atlys board. If we increase the length of the correlations modules, starting from 20 and up to 400, the amount of required logic gates remains almost constant (Figure 13).

In the case of one transmitter, there are a few drops noticeable in the slice occupation. This can be explained since at these points, the length of the correlations module *m* exceeds a power of two, and some optimizations are possible. When extending this implementation to four transmitters, these optimizations are not possible any more. Furthermore, the amount of slices, LUTs and registers increase when the number of transmitters is increased to four and remain below 30%. Furthermore, here, all of the samples are stored in 63 BRAM modules.

## 6. Ranging Analysis

In our analysis, we compare the two proposed ranging methods. In the first set of analyses, we estimate the distance using the first implementation case. This enables us to better estimate the correlator efficiency. However, this approach only enables a single transmitter single receiver topology. In the second set of analysis, the second method using the ADC and FIR filtering is implemented. This second topology allows one to implement a system where multiple transmitters may simultaneously send ultrasonic coded signals while the receiver is capable of discriminating the different signals (*i.e*., multiple access). In this set, we investigate the efficiency of the receiver by first estimating the distance against one transmitter. Afterwards, two transmitters are used in order to estimate the robustness of our implementation against the near-far phenomenon.

A few aspects need to be taken into account while applying the ranging analysis. The first aspect concerns the amount of samples *versus* the distance. As the distance *d* between the transmitter and receiver increases, the time difference between the correlation peak and the synchronization increases. This time difference is expressed as a linear relationship against the distance (*d*) between transmitter and receiver and the amount of samples. This relationship is given in Equation (8), where *α* (*i.e*., slope) and *c* (*i.e*., offset) are calculated through regression analysis.

(8) d=α·samples+c

A second aspect of concern is the attainable precision and accuracy. In ideal circumstances, the theoretical maximum precision is given by the component with the lowest precision. Components affecting the precision are the correlator and the signal conditioning (*i.e*., analog-to-digital conversion speed rate). The highest attainable precision is given in Equation (9), where the “Min” function takes the lowest value. In the case that the correlator length matches (Equation (4)) the sampling frequency, both the correlator and analog-to-digital conversion result in the same level of precision. The attainable precision *p* is determined by:

(9) $$p=\frac{v_{sound}}{Min(f_{s},m\cdot B)}$$

with $v_{sound}$ the speed of sound and $f_{s}$ the sampling frequency. The precision also corresponds to the minimal distance step between two consecutive samples (*i.e*., the slope *α* in Equation (8)).

Another aspect regarding the measurements concerns the correlation quality. The correlator compares the ultrasonic signal against a given orthogonal code. The positive correlation result is bounded between zero and 100% and is therefore used as a metric to estimate the quality of the measurement. The last aspect that influences the measurements is the piezoelectric crystal used at the transmitter side. This crystal suffers from inertia when a frequency shift occurs. This phenomenon causes a signal degradation during the first 10 to 20 cycles when a new frequency is applied. To compensate this inconvenience, a lower baud rate (*i.e*., frequency shift rate) than proposed in a previous work is chosen [9].

All measurements are repeated 250 times. This enables us to detect faulty measurements. The Gaussian distribution is applied to calculate the 95% confidence interval, and the average of each 250 measurements is taken and compared to the real distance between transmitter and receiver. This gives an estimation of the accuracy. In the first setup, measurements are taken at an interval of 20 cm, ranging from 0 cm up to a distance of 9.00 m. In the other setups, the measurements are taken at an interval of 40 cm, starting at a distance of 0 cm and ranging up to a distance of 17.20 m. The range of 17.20 m corresponds to the diagonal of the largest room available at our disposal. In a final experiment, the location of the receiver is estimated by using the trilateration approach.

The orthogonal codes are used to provide the ability to distinguish different transmitters at the receiver side. At the transmitter side, the code is used to generate the appropriate carrier frequency. In our implementations, we use a Gold code of 63 symbols. Each of the symbols specifies which of the carrier frequencies is used. Due to the narrow bandwidth of the piezocrystal transducer, we choose 24.5 kHz and 26 kHz as carrier frequencies around the resonance frequency (*i.e*., 25 kHz) of the transducer. Both frequencies also delivered the best correlation results during preliminary tests.

### 6.1. Single Transmitter, Single Access Receiver Implementation

**Figure 14:**
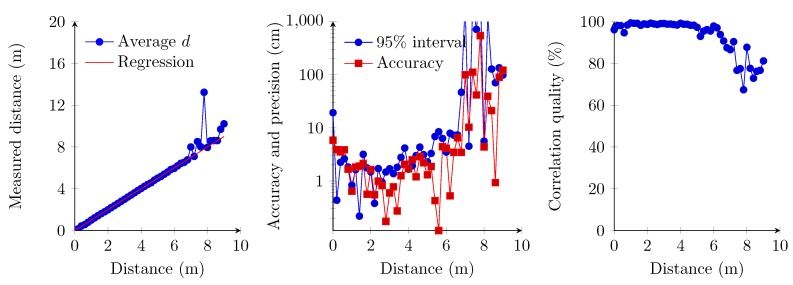
Linear regression of the measured distances (**left**); accuracy and the 95% confidence interval (**middle**); and correlation quality (**right**).

The first set of measurements concerns the implementation where the receiver is implemented using the first demodulation topology and where the sampling frequency of the frequency finder is set at 25 kSps. This sampling frequency therefore results in a maximum attainable precision of 1.4 cm (Equation (9) at 20 ${}^{\circ }$C). The experiment shows a linear relationship between the measured distance and the distance from 0 cm up to 6.60 m (Figure 14). However, the 95% confidence interval (Figure 14) indicates a less precise measurement for the 0-cm distance, which accords with a previous work [9]. The 95% confidence interval and correlation quality strongly deteriorate at a distance beyond 6.60 m (Figure 14). Therefore, the zero distance and values beyond the 6.60-m boundary are omitted in the regression analysis. One should also note that correlation quality, accuracy and precision remain constant within the interval of 20 cm and 6.60 m. The precision obtained is in the best case comparable to the maximum attainable precision, which corresponds to the slope obtained with the regression analysis.

During these measurements, we also observed abnormalities beyond the 6.60-m boundary. We measured several times a positive offset of approximately 2 m compared to the real distance. The correlation quality at these measurements also decreased drastically below 70%. This constant offset could be caused by autocorrelation due to a lower ultrasonic signal quality.

### 6.2. Single Transmitter at 3.3 V, Multiple Access Receiver Implementation

**Figure 15:**
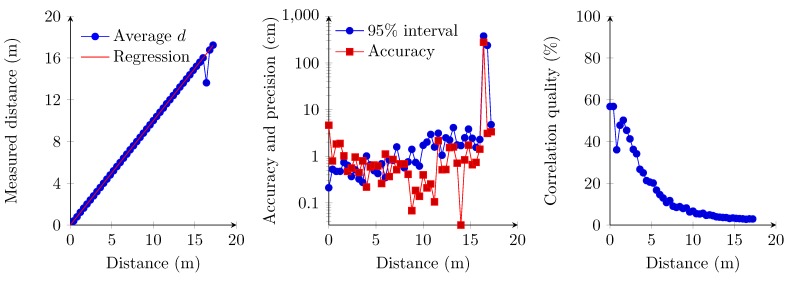
Linear regression of the measured distances (**left**); accuracy and the 95% confidence interval (**middle**); and correlation quality (**right**). In these measurements, the amplitude of the transmitter is 3.3 V in a single access topology.

These measurements concern the implementation where the receiver supports multiple simultaneous transmissions from several transmitters. In this setup, a sampling frequency of 100 kHz is chosen, leading to a maximum precision of 3.43 mm (Equation (9) at 20 ${}^{\circ }$C). We use a single transmitter that emits ultrasound signals with the transducer set at a 3.3-V amplitude. The experiment shows a linear relationship between the measured distance and the distance from 0 cm up to 17.20 m (Figure 15). In these measurements, the 95% confidence interval (Figure 15) again indicates a less precise measurement for the 0-cm distance. The 95% confidence interval and accuracy strongly deteriorate at distances beyond 16.00 m (Figure 15). Therefore, the zero distance and values beyond the 16.00-m boundary are omitted in the regression analysis. The slope of the regression analysis (Equation (8)) is also quite close to the predicted maximum precision value (Equation (10)).

(10) d=0.34542·samples-8726.26

The correlation quality in Figure 15 drops exponentially. This is due to the sound intensity, which is inversely proportional to the square of the distance from the sound source.

### 6.3. Single Transmitter at 12 V, Multiple Access Receiver Implementation

**Figure 16:**
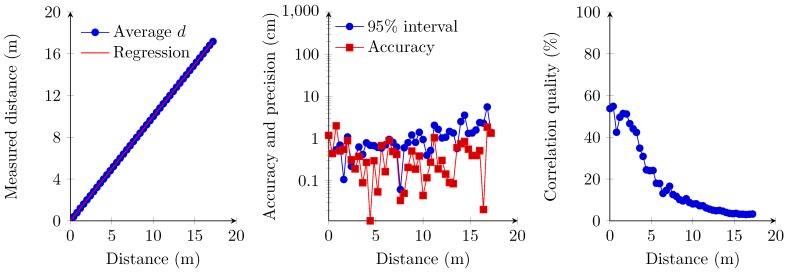
Linear regression of the measured distances (**left**); accuracy and the 95% confidence interval (**middle**); and correlation quality (**right**). In these measurements, the amplitude of the transmitter is 12 V in a single access topology.

These measurements also concern the implementation where the receiver supports multiple simultaneous transmitters. Compared to the previous set of measurements, the only difference is the transmitters’ transducer amplitude, which is set at 12 V. However, the amplitude of 12 V on the transmitters’ transducer is only applied in order to compare against the amplitude of 3.3 V from the previous set of measurements. Since the FPGA is already fed by a 3.3-V power supply, 12 V requires more hardware (*i.e*., level shifters) to interface the transducer together with an additional power supply. The same sample frequency as in the 3.3-V setup is used, and thus, the same level of precision can be obtained. The experiment shows a linear relationship between the measured distance and the distance from 0 cm to 17.20 m (Figure 16). The 95% confidence interval (Figure 16) indicates more precise measurements for the 0-cm distance. For the other distances, the values of the 95% confidence interval slightly decrease to 5.6 cm when the distance between the transmitter and the receiver ranges beyond 16 m. Due to the higher transmission power of the transmitter, the accuracy remains stabler at distances between 16.00 and 17.20 m. The slope of the regression analysis (Equation (8)) is also quite close to the predicted maximum precision value (Equation (11)).

(11) d=0.34565·samples-8732.99

The correlation quality trend (Figure 16) is similar to the correlation quality trend obtained in the previous section (Figure 15).

### 6.4. Multiple Transmitter, Multiple Access Receiver Implementation

**Figure 17:**
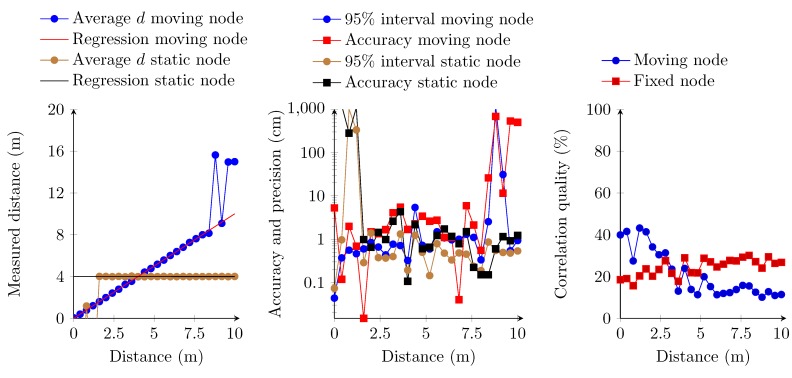
Linear regression of the measured distances (**left**); accuracy and the 95% confidence interval (**middle**); and correlation quality (**right**). In these measurements, the amplitude of the transmitters equals 3.3 V and is put into a multiple access topology.

In these last series of measurements, we estimate the distance between two transmitters against one receiver. The transmitters modulate the ultrasound signals using an amplitude of 3.3 V. An amplitude of 12 V is also possible, but this requires additional hardware and power supplies. Therefore, we only measure the distance with the transducers set at an amplitude of 3.3 V. The receiver and the first transmitter are set at a fixed distance of 4 m. The second transmitter is set at increasing distances from the receiver, with intervals of 40 cm and ranging from 0 cm up to 10 m. Both transmitters are transmitting their respective orthogonal sequence simultaneously and, thus, interfere with each other. Results show that measuring the distance between both transmitters and the receiver is possible from 1.6 m up to 8 m (Figure 17). The fixed transmitter is detected when the second transmitter is at approximately 1.6 m from the receiver. In the opposite way, the range between the second transmitter and the receiver can be obtained up to a distance of 8 m. The overall distance accuracy and precision remain both at the centimeter level in the 1.6 m to 8 m range. The correlation quality of both signals, however, drops compared to previous measurements. This is due to the interference caused by both transmitters. The correlation quality of the fixed transmitter augments following an asymptotic curve when the other transmitter is moved from the receiver. By contrast, the correlation quality of the moving transmitter follows the same trend as in previous multiple access implementations. One should also remark that the correlation quality of both transmitters does not decrease below a threshold of 10%. This can be due to interference, which contaminates the correlation of both transmitters. The problem of detecting only the nearest transmitter is also known as the near-far problem within CDMA.

All of the measurements show a strong relationship between the measured distance and the real distance between transmitters and receiver. In the first set of measurements, the receivers’ hardware digitizes the analog signal into a logic “1” or “0”. This reduces the amount of required FPGA logic. However, this removes the receivers’ ability to identify two simultaneous transmitters. This digitization at the hardware level enables one to have a correlation quality of almost 100% for short distances. This correlation suddenly drops beyond a distance of 6.60 m. At this same distance, the accuracy and precision of the measurements also strongly deteriorate. This drop is mainly due to the signal degradation, which causes the hardware to generate random ones and zeros instead of a periodic signal. The latter also affects the attainable distance between transmitter and receiver. In all other measurements, the correlation quality decreases exponentially. Here, the hardware delivers an analog signal, which is digitized by means of an ADC. As such, the system still detects the signal until it reaches the noise floor of the MEMS microphone. This enables the system to measure distances beyond 15 m in single access mode and up to 8 m in multiple access mode. In these measurements, the correlation quality does not exceed a threshold of 60%. This threshold is due to the signal conditioning modules, which decreases the signal amplitude during the filtering, by taking the absolute values of the signal and especially by averaging the signal for each frequency channel. The signal averaging reduces the signal amplitude to a theoretical maximum of 70.7% of its amplitude. Other factors, like the hardware, may also slightly influence the signal quality.

### 6.5. Indoor Localization

**Figure 18:**
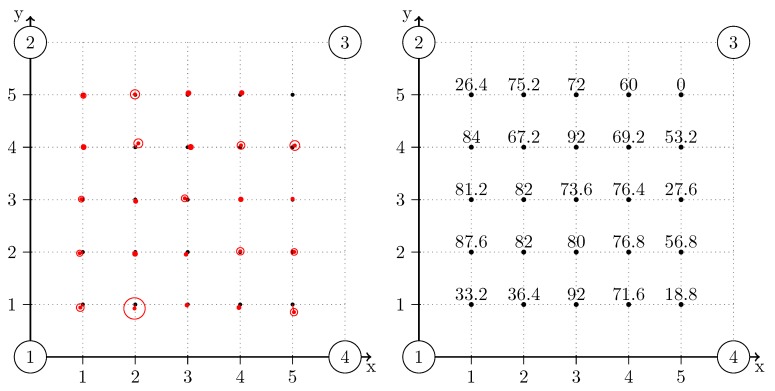
Accuracy and precision of the trilateration process at each point of the grid (**left**); The ratio of successful trilateration attempts (**right**). A total of 250 attempts were issued for each location on the grid. Transmitters are represented by a big circle with the corresponding ID.

**Figure 19:**
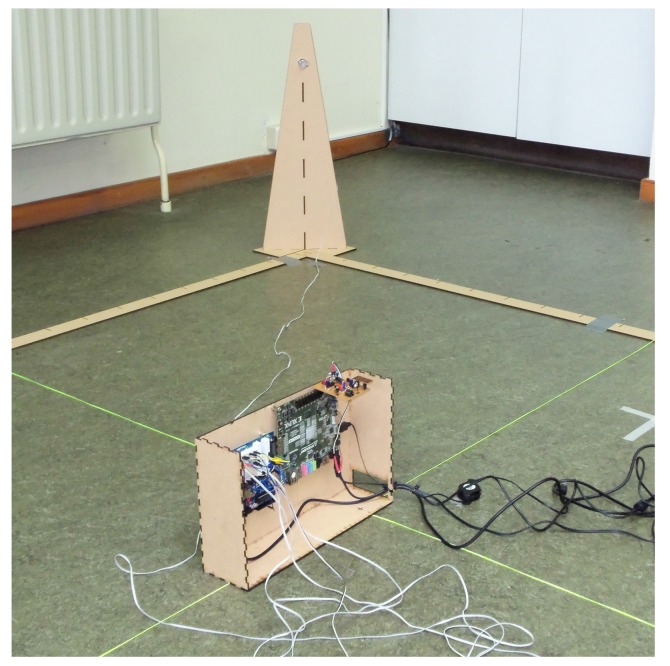
Test setup of the indoor localization implementation showing the receiver and one transmitter in one corner of the map.

The ultimate goal of our implementation is to allow a device to retrieve its current position in a room. In order to demonstrate the feasibility of our system, we also apply indoor localization analysis. We build a grid of 6 m by 6 m, with four transmitters placed at each corner of the grid. The receiver is placed at intervals of 1 m onto the grid. The setup is depicted in Figure 18, where each measurement point is shown with a black thick dot. Figure 19 depicts the setup equipment in our lab. All transmitters send their corresponding ultrasound signal at a random interval. The position of the receiver is calculated with the following steps:

1. Each distance estimation against all transmitters is calculated.
2. The three closest positive distances are kept.
3. 2D trilateration is calculated with these three measurements by calculating the intersection point of three circles. The Gaussian elimination is used during this process. A 2D position is obtained from this process.
4. The average and the 95% confidence interval are calculated following the Gaussian distribution. During this stage, outliers are eliminated. These outliers include the positions that are outside the grid and the positions that are outside the 95% confidence interval.
5. The average position and the 95% confidence interval are recalculated on the remaining positions, and the amount of successful trilateration attempts is also calculated.

The measurements are applied by taking 250 distance measurements against each transmitter at each location on the grid. The relative amount of successful trilateration attempts is shown in Figure 18. A higher amount of successful attempts indicates a more reliable localization.

Results show a tendency of a higher reliability of localization when the receiver is located towards the center of the grid. At each corner of the grid, the amount of successful attempts drops, and at position (5,5), the location of the receiver could not be retrieved. This phenomenon depicts the near-far problem previously mentioned in this paper.

The accuracy and precision are represented, respectively, by a red dot and a red circle on the map. The accuracy and precision generally remain below 5 cm.

## 7. Conclusions

In this paper, we proposed a novel implementation approach for ultrasound orthogonal ranging towards indoor localization using FPGAs. The implemented system is composed of one or two transmitters that are sending ultrasound signals using conventional piezoelectric transducers. On the receiver side, a MEMS transducer converts the ultrasound signals into electrical signals. These signals are processed by the FPGA in order to apply range estimation. The ultrasound signals are composed of FSK-modulated signals, where the carrier frequency sequences are specified by orthogonal Gold codes. The FPGA demodulation on the receiver side consists of a conditioning module followed by the correlation module. The demodulator allows one to track up to four transmitters simultaneously, while keeping the required FPGA resources below a threshold of 30%. Several ranging experiments have been set up, including the retrieval of the location of the receiver on a map. In the single transmitter ranging measurements, the maximal attainable distance depends on the receivers’ topology and on the signal amplitude emitted by the transmitter, where a distance range of more than 10 m could be obtained. When two transmitters are sending simultaneously, this range decreases to approximately 8 m. This limitation is due to the near-far problem. This limitation could also be verified when attempting to retrieve the location of the receiver on a map. However, the obtained ranges allow one to cover small to medium-sized rooms. All of these advantages together allow the deployment of an indoor localization system by implementing the computational requirements of the orthogonal signals’ demodulation and, thus, by proposing a more noise-robust system, on a low-power embedded system. The system however utilizes a wire to synchronize the transmitters with the receiver. In real-world implementations, one expects a wireless device for indoor localization. Our system also implements the position retrieval based on the Gaussian elimination scheme. For real-world applications these shortcomings need to be alleviated. Although the ranges proposed in this work allow one to apply ranging to small to medium-sized rooms, a combination of a radio frequency (RF)-based synchronization and smart trilateration algorithms based on statistical approaches is necessary to augment the reliability towards indoor localization.
